# Predicting how evolution will beat us

**DOI:** 10.1111/1751-7915.13327

**Published:** 2018-11-20

**Authors:** Tom Ellis

**Affiliations:** ^1^ Department of Bioengineering Imperial College London London SW7 2AZ UK; ^2^ Centre for Synthetic Biology Imperial College London London SW7 2AZ UK

## Abstract

In this Crystal Ball article, we look ahead to the possibility of model and tools that can predict the accumulation of mutations that inactivate engineered plasmids.

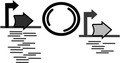

Most biotechnological applications of microbiology have well‐characterized cells growing in defined and stable environments. Yet even in such idyllic conditions, we fear the two powers of evolution: mutation and selection Why? Because they consistently take aim at our own engineering, inactivating the new genes we have introduced into cells and then growing faster and overtaking our productive population. However, given everything we now know about microbial molecular biology, biotechnology and genomics, we should soon be at the point where we can predict how these two forces will play out.

For predicting evolution, our best bet is to consider how *Escherichia coli* engineered with plasmids end up being outcompeted by cells that acquire inactivating mutations. Plasmid‐based gene expression is widely used in research and biotechnology and is the most common form of genetic engineering used today. Compared to integrating genes into the host genome, it is an inelegant approach and prone to problems, yet remains the go‐to‐method for the overproduction of proteins and for engineering bacterial cells for biosynthesis of metabolites. Thanks to the failures of thousands, there are documented (and countless undocumented) cases where populations of *E. coli* transformed with plasmids have evolved to inactivate the expression of heterologous plasmid‐hosted genes. How quickly this evolution occurs is defined by the strength of two powers of evolution: the likelihood of mutation and the degree of selection.

While predicting likely mutations sounds like a fantasy for those who study evolution of organisms and genomes, it is actually not so far‐fetched when we narrow things down to just the few thousands of bases that comprise engineered plasmids in *E. coli*. Most loss‐of‐function mutations in cells engineered via plasmid transformation are likely to be mutations to the plasmid, not the genome, and substantial research tells us that the plasmids typically used in biotechnology have common forms of mutation (Oliveira *et al*., 2009). In particular, deletions due to intra‐plasmid recombination are common, removing out entire chunks of the plasmid at a time. Another type of mutation is caused by the transposable ‘insertion sequence’ (IS) elements which insert themselves at poorly conserved sequence motifs. *E. coli* has a diversity of IS elements with varying specificity, and these are well known to disrupt plasmids at times of stress by transposing into permissive sites.

Two key synthetic biology studies from the past decade have given us some much‐needed characterization data on the escape mutations that lead to plasmid‐burdened *E. coli* being outcompeted. Sleight *et al*. used modular DNA assembly to build a library of different plasmids expressing three different fluorescent proteins from different promoters and followed the loss of fluorescence over time as the *E. coli* hosting these plasmids were passaged over several days in the laboratory (Sleight and Sauro, 2013). Traditional sequencing was then used to reveal the types of mutations that inactivated expression. Recombination‐mediated deletion was found to be the main culprit in these designs. More recently Rugbjerg *et al*. (2018) used next generation sequencing to track the different escape mutants that led to loss of productivity in *E. coli* engineered with a plasmid encoding a metabolic biosynthesis pathway. Interestingly, no obvious mutations were identified on the host genome that led to loss of productivity and faster cell growth, instead escape mutations were only ever found on the plasmid, and in this case were typically caused by IS elements.

To help predict plasmid‐based mutation in *E. coli*, the Barrick laboratory devised the ‘Evolutionary Failure Mode (EFM) Calculator’ in 2015 (Jack *et al*., 2015). This easy‐to‐use software tool analyses a plasmid DNA sequence file, searching for homologous sequences found multiple times in each plasmid (which are hotspots for recombination‐mediated deletion) and simple sequence repeats (which are hotspots for short insertions and deletions). Based on the occurrence of these, it will give a plasmid a relative instability prediction (RIP) score: the higher the RIP, the higher the chance that the plasmid will mutate. The current version does not consider mutation caused by IS elements, but one can expect that with further work an updated calculator could be developed to also take into account the most prevalent IS elements from the most commonly‐used strains of *E. coli*. If this and consideration of the underlying rate of mutation of plasmid sequences (*i.e*. the infidelity of DNA replication) could be added to the calculator, then we would essentially have a prediction tool for plasmid mutation.

Of course, mutation is only one of the two powers of evolution; so what about selection? For *E. coli* growing in large populations in defined and stable environments, selection is effectively equal to growth rate. A cell growing slower than the rest of the population will eventually be diluted out and lost, with the speed of loss directionally proportional to how much slower its growth is compared to the rest. What makes a cell with an engineered plasmid grow slower than its rivals is burden, a long talked‐about problem that has recently seen renewed interest.

Burden comes in three flavours. Firstly, just the basic cost of expressing any extra RNA or protein is a burden, as it takes up energy, machinery and resources to do so (*expression burden*). Secondly, an RNA or protein expressed out of context may have an intended function that consumes key cellular resources, such as an enzyme that produces a natural product from host metabolites (*role‐based burden*). Finally, the RNA or protein may have unintended interactions with processes in the cell that impair them, such as a membrane protein that aggregates with the host's transport channels (*toxicity burden*). Of these, only expression burden is universal and recent studies have shown that expression burden from plasmids in *E. coli* can be considerable. This means that even if a protein does nothing destructive inside a cell, just the cost of making a lot of it can severely slow growth.

Again, thanks to recent work in synthetic biology, we now have ways to quantify and predict the burden of gene expression, and these do remarkably well at estimating how *E. coli* growth rates decrease when these genes are expressed from plasmids (Ceroni *et al*., 2015). Protein‐encoding genes can now be measured for burden by a standard *in vitro* method ahead of any cloning, and these data then used to predict what the growth rate decrease will be when this is used in a multigene constructs (Borkowski *et al*., 2018). As growth rate is the selection pressure in the typical conditions of industrial use of *E. coli*, the burden prediction methods effectively define the power of selection against all genes that do not have significant role‐based or toxicity burdens.

So perhaps it is now time to put these efforts together. We can predict the likely mutations of common plasmids in *E. coli*, and we can measure genes and then predict the selection pressure against them. It follows that when a plasmid is designed, we could make probabilistic models of how it could mutate and assign scores for how much each mutation would decrease the burden (*i.e*. improve selection). This could then be used to estimate which escape mutant would likely dominate a population in an experiment, and how many hours or generations it would take for this to happen. This would not be a deterministic prediction as mutation is inherently stochastic, but randomness would be smoothed by the large numbers of cells of *E. coli* in most experiments.

We should not forget as well that plasmids are usually present at multiple copies per cell too, and while this reduces noise, it adds in further complexity. How does the growth rate of a cell change when just a fraction of the plasmids have a mutation, and how much of a role does plasmid partitioning have in propagating mutations? To understand these issues, we could certainly do with more data and ideally single cell data too. Flow cytometry, microfluidics microscopy and even single cell sequencing all afford ways to provide this, allowing us to see how escape mutations in plasmids accumulate in cells, and then enabling us to better predict how these mutations spread through a population.

Having a plasmid evolution prediction tool such as this would likely be broadly useful for both research and biotechnology. In the first instances, it would allow us to be more realistic about how long we can expect our genetic engineering to hold out for; for high‐burden plasmids in fast competitive growth, this may well be never more than a day or two. Understanding these limitations from the outset would encourage more development and uptake of alternative engineering approaches like genomic integration of expression cassettes or feedback mechanisms that prevent toxic overexpression.

Such a tool would also predict what the likely mutants may be that will emerge from long‐term uses of engineered cells, such as when modified *E. coli* are placed in the gut microbiome for months at a time. Plasmids could also be intentionally designed to break in predicted ways over time as a novel route to implement long‐term dynamic changes in how engineered cells behave. A further benefit of any prediction tool is that it can help reveal gaps in knowledge. Where prediction and experimental outcomes fail to match, there is usually an opportunity to uncover new information that can identify important mechanisms not previously considered.

Most importantly, a plasmid evolution prediction tool would allow those designing plasmids to reconsider bad designs ahead of experiments. Designers could lower expression levels wherever possible to reduce expected burden in order to delay inactivation by mutation. They can also ensure removal of the sequence features most likely to promote mutation and consider changing hosts to those that are free from certain IS elements if these are expected to be a main source of mutation. Ultimately, being better genetic designers is what synthetic biologist strive to be, and even if it is an unsurmountable task to fight evolution, it would be prudent to at least be designing with it in mind.
